# The microbial nitrogen cycling potential is impacted by polyaromatic hydrocarbon pollution of marine sediments

**DOI:** 10.3389/fmicb.2014.00108

**Published:** 2014-03-25

**Authors:** Nicole M. Scott, Matthias Hess, Nick J. Bouskill, Olivia U. Mason, Janet K. Jansson, Jack A. Gilbert

**Affiliations:** ^1^Institute of Genomic and Systems Biology, Argonne National LaboratoryLemont, IL, USA; ^2^Department of Ecology and Evolutionary Biology, University of ChicagoChicago, IL, USA; ^3^Energy and Efficiency Division, Chemical and Biological Process Development Group, Pacific Northwest National LaboratoryRichland, WA, USA; ^4^Systems Microbiology and Biotechnology Group, Washington State UniversityRichland, WA, USA; ^5^Ecology Department, Earth Sciences Division, Lawrence Berkeley National LaboratoryBerkeley, CA, USA; ^6^Earth, Ocean and Atmospheric Science, Florida State UniversityTallahassee, FL, USA

**Keywords:** nitrogen cycling, marine sediments, denitrification, microbial ecology, metagenomics, deepwater horizon oil spill, oil contamination, oil seeps

## Abstract

During hydrocarbon exposure, the composition and functional dynamics of marine microbial communities are altered, favoring bacteria that can utilize this rich carbon source. Initial exposure of high levels of hydrocarbons in aerobic surface sediments can enrich growth of heterotrophic microorganisms having hydrocarbon degradation capacity. As a result, there can be a localized reduction in oxygen potential within the surface layer of marine sediments causing anaerobic zones. We hypothesized that increasing exposure to elevated hydrocarbon concentrations would positively correlate with an increase in denitrification processes and the net accumulation of dinitrogen. This hypothesis was tested by comparing the relative abundance of genes associated with nitrogen metabolism and nitrogen cycling identified in 6 metagenomes from sediments contaminated by polyaromatic hydrocarbons from the Deepwater Horizon (DWH) oil spill in the Gulf of Mexico, and 3 metagenomes from sediments associated with natural oil seeps in the Santa Barbara Channel. An additional 8 metagenomes from uncontaminated sediments from the Gulf of Mexico were analyzed for comparison. We predicted relative changes in metabolite turnover as a function of the differential microbial gene abundances, which showed predicted accumulation of metabolites associated with denitrification processes, including anammox, in the contaminated samples compared to uncontaminated sediments, with the magnitude of this change being positively correlated to the hydrocarbon concentration and exposure duration. These data highlight the potential impact of hydrocarbon inputs on N cycling processes in marine sediments and provide information relevant for system scale models of nitrogen metabolism in affected ecosystems.

## Introduction

Petroleum hydrocarbon spills considerably alter the composition and functional dynamics of marine microbial communities (Hazen et al., 2010; Gutierrez et al., 2013). Ultimately, microorganisms that can respond to complex hydrocarbon mixtures are preferentially enriched by hydrocarbons provided during an oil spill; growing from small natural seed populations (Röling et al., 2002, 2004). A number of studies have demonstrated changes in the spatiotemporal ecology of microbial communities in the presence of oil contamination (Bordenave et al., 2007; Paissé et al., 2008; Hazen et al., 2010; Bælum et al., 2012; Lu et al., 2012; Mason et al., 2012), which has concomitant impacts on microbially-mediated biogeochemistry (Atlas, 1991). Proliferation of heterotrophs in the presence of hydrocarbons as the sole electron donor can result in the rapid depletion of oxygen in the ecosystem through aerobic respiration (Riser-Roberts, 1992).

One potential limiting factor for hydrocarbon degradation in marine sediments is nitrogen availability (Herbert, 1999). Marine sediments are a key location for global nitrogen cycling (Engström et al., 2009; Lam and Kuypers, 2011; Hamme and Emerson, 2013), because they provide both a physical location for organic matter remineralization through ammonification, and also an anoxic environment for denitrification processes (Laverock et al., 2011). At the water-sediment interface, ammonium generated through organic matter remineralization, is converted to nitrite and then nitrate by nitrification (Henriksen and Kemp, 1988). Available nitrite or nitrate may diffuse to the water column or be consumed in various biotic reactions depending on oxygen availability. Under oxygen limited conditions, deeper within the sediment, nitrate may be reduced via dissimilatory nitrate reduction to ammonium (DNRA), which can be anaerobically oxidized, with nitrite, to dinitrogen, through anammox processes, also the experimental evidence for this mechanism remains limited (Brandes et al., 2007; Kalvelage et al., 2011). It has been estimated that dinitrogen production in deep benthic sediments (1000–3000 m) is responsible for ~7–16% of the total nitrogen loss in the marine ecosystem (Engström et al., 2009).

How oil contamination affects nitrogen cycling processes *in situ* is still not well understood (Deni and Penninckx, 1999; Bell et al., 2011; Trimmer et al., 2013). Although petroleum oil is typically rich in nitrogen, most of it is bound in aromatic heterocyclic compounds whose carbon to nitrogen bonds are difficult to break (Snyder, 1970). Thus, bioremediation of oil contamination often requires the addition of inorganic nutrients including nitrogen, phosphorus, and/or iron, to increase enzyme activity (Brook et al., 2001; Head et al., 2006; Bell et al., 2011). In the case of high carbon and low nitrogen environments, there is evidence of increased diazotrophy within microbial communities (Karl et al., 2002), although this has rarely been observed in the presence of hydrocarbons despite nitrogen limiting conditions (Laguerre et al., 1987; Eckford et al., 2002; Musat et al., 2006). A more recent study exploring the relationship between microbial nitrogen cycling dynamics and oil contamination found evidence of DNRA being coupled to nitrogen cycling in a suboxic hydrocarbon contaminated subsurface well (Yagi et al., 2010).

A number of studies have evaluated how well the relative abundance of genes encoding nitrogen metabolic enzymes correlate with biogeochemical measurements of N metabolism. For instance, the abundance of *nirS, nrfA, narG*, and *napA* genes involved in nitrite and nitrate reduction significantly predicted the rates of denitrification and DNRA in the Colne estuary (Dong et al., 2009). However, *nirK*, another nitrite reduction gene, has been shown to be a poor predictor of functional traits relevant for denitrification (Salles et al., 2012). The metabolic pathways themselves are a network of interactions, so we hypothesize that the non-linear relationship between gene abundance and metabolite turnover is best evaluated as a function of compound changes in the relative abundance of many different related genes, rather than any single gene abundance. Predicted relative metabolic turnover (PRMT) quantifies relative changes in the metabolic potential as a network of predicted metabolic reactions inferred from the relative abundance of genes annotated from a metagenome (Larsen et al., 2011). Each predicted metabolite is then a function of the predicted enzymes and their “metabolic community of reactions” rather than simply the relative abundance of just the single gene that codes for the enzyme responsible for the metabolism. PRMT has previously been used to accurately predict seasonal variation in metabolites in marine surface waters (Larsen et al., 2011).

Here we hypothesize that increasing exposure to elevated hydrocarbon concentrations will positively correlate with predicted metabolic shifts toward denitrification in anaerobic zones in sediments. This is based on the premise that previously challenged and constantly exposed hydrocarbon samples are more likely to be “primed” for hydrocarbon response (Deni and Penninckx, 1999; Labbé et al., 2007; Taketani et al., 2010). To test this hypothesis we analyzed metagenomic sequence data from 17 sediment samples from the Gulf of Mexico and the Santa Barbara Channel, which represent sites with short term exposure to oil contamination [those from the Deepwater Horizon (DWH) oil spill], sites with a long history of exposure to hydrocarbons (those from the natural oil seeps), and sites unaffected by hydrocarbon contamination.

## Materials and methods

### Data

Data were downloaded from MG-RAST (Meyer et al., 2008) including the DWH spill project (MGRAST IDs: 4510162.3-4510175.3) and the natural oil seeps study (MGRAST IDs: 4537092.3-4537094.3), which were all annotated using SEED, with a maximum *e*-value of 1 × 10^−3^, a minimum identity of 50%, and minimum identity cutoff of 15. Data was also annotated in MG-RAST using Hierarchical Classification subsystems with a maximum *e*-value cutoff of 10^−5^, minimum percent identity cutoff of 60%, and a minimum alignment length cutoff of 15; this data was used for looking at functional annotations for gene abundances. For the samples collected from the Gulf of Mexico during the DWH spill, 6 of the samples were from oil-contaminated sites (hereafter referred to as DWH oil spill samples), and 8 samples were from uncontaminated sites (hereafter referred to as uncontaminated samples). This grouping was based on whether the samples clustered based on the normalized gene abundances, and additionally based on whether they exceeded (>1.0 polycyclic aromatic hydrocarbon [PAH] index) or did not exceed EPA (≤1 PAH index) BPA benchmarks for hydrocarbon pollution (Mason et al., 2014) (for more information about how the EPA aquatic benchmarks are calculated please see http://www.epa.gov/bpspill/water-benchmarks.html#dblstar). The Santa Barbara channel oil seep samples included depth, latitude and longitude, and collection date as contextual metadata. This data is summarized in Supplementary Table 1. The Gulf of Mexico samples (Mason et al., 2014) had these contextual metadata in addition to total petroleum hydrocarbons (TPH), polycyclic aromatic hydrocarbons (PAH), dissolved-phosphate (PO_4_-P), dissolved nitrate (NO_3_-N), total ammonia nitrogen (NH_3_-N and NH_4_-N), dissolved inorganic nitrogen (DIN; NH_3_-N and NH_4_-N), total nitrogen (NH_3_/NH_4_-N, NO_3_/NO_2_-N, and organic nitrogen), total sulfur (S), and total carbon (C). A complete metadata table for the Gulf of Mexico samples is given in Supplementary Table 1 of Mason et al. (2014). For more information about sample collection and the context of these samples please see Hawley et al. (2014), Mason et al. (2014). These values were normalized and log_2_ transformed before analysis was performed.

### Analysis

The oil seep samples had genetic sequences that annotated to 131 nitrogen metabolism genes that were not present in any of the Gulf samples. Thus for gene annotations, SEED Subsystems-based functional (level 2) annotations were summed and then standardized as a function of total reads within each sample. Predicted Metabolic Turnover Analysis (PRMT) (Larsen et al., 2011) was used to evaluate the community metabolic potential between samples as a function of microbial community gene abundances. PRMT transforms annotated enzyme abundances by a weighted matrix of all possible reactions including those enzymes, their reactions, and associated metabolites as annotated by KEGG (Ogata et al., 1999). Enzyme commission (EC) abundances were gathered from the SEED Subsystems L3 tables, quantile normalized and then log_2_ transformed before analysis. The EC abundances were compared to a “reference,” which in this analysis was an average of all samples. Positive PRMT score values represent the consumption of a particular metabolite, and negative scores represent the accumulation or production of a particular metabolite. For the nitrogen metabolism pathway (KEGG map00910), the PRMT scores were summed to give either a “net” positive or negative PRMT value. The “net difference” or “pathway flow” was found by adding the net positive and net negative values for PRMT scores for each metabolite in the pathway per sample. For comparisons of sample scores, Kruskal-Wallis rank sum tests were used. Hierarchical annotation for gene abundances were also quantile normalized and log_2_ transformed. A principal component analysis was performed on the quantile normalized and log_2_ transformed hierarchical abundances, removing those genes completely absent in the Gulf of Mexico dataset. To pull out hydrazine related gene sequences, bowtie 1.0.0 (Langmead et al., 2009) was used to align reads to custom index of hydrazine hydratase related sequences downloaded from NCBI (Benson et al., 2013). Then we used reads per kilobase per million (RPKM), quantile normalization, and a log_2_ transformation to normalize the hydrazine hydratase related gene abundances. Pearson correlation coefficients (corr) were used where cited. 10,000 permutations were used to assess empirical *p*-values.

## Results

The contaminated sediments from the Gulf of Mexico were exposed to hydrocarbon contamination from the DWH spill for between 3 and 5 months at the time they were collected. By contrast, the natural oil seeps from the Santa Barbara Channel were estimated to be exposed to hydrocarbons for more than 11,000 years (Hornafius et al., 1999). To the best of our knowledge the uncontaminated sediments from the Gulf of Mexico had not recently been exposed to the amounts of hydrocarbon contamination caused by the DWH Oil spill, although historical presence of temporary natural oil seeps nearby cannot be ruled out (Kvenvolden and Cooper, 2003). The metagenomic data were all generated using the HiSeq2000 platform, with a minimum of 36,851,796 reads and a maximum of 86,321,188 reads per metagenome, with read lengths of ~150 bp.

### Oil seep samples maintained a greater diversity of genes associated with nitrogen metabolism

Oil seep samples had sequences that annotated to 131 nitrogen metabolism genes; these genes were not present in the samples from the Gulf of Mexico (oil spill and uncontaminated sites). Of the 11 SEED level 2 annotations within nitrogen metabolism, 4 were present only in the oil seep samples- these included amidase clustered with urea and nitrile hydratase functions, cyanate hydrolysis, citric oxide synthase, and nitrilase.

### Nitrogen metabolism genes showed differential relative abundances between the 3 different sample groups

**Figure 1:**
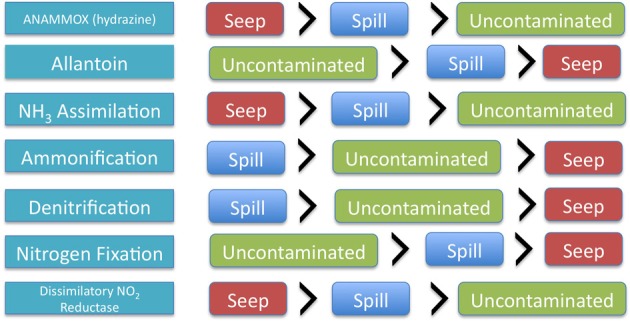
**Graphical representation of the changing relative abundance of different key nitrogen pathways and genes within each of the sample types**.

Anammox pathway specific genes related to hydrazine production (an intermediate of the anammox reaction pathway) were not significantly different between oil seep, oil spill and uncontaminated samples; however they trended toward higher abundance in oil seep samples, followed by oil spill samples and uncontaminated samples. Interestingly, nitrosative stress, was found only in the petroleum-contaminated groups (both oil spill and oil seep), and is involved in response to nitric oxide accumulation (Ridnour et al., 2004). Coincidentally, nitric oxide is predicted to be accumulated by the PRMT analysis (Table 2). Interestingly, only 3 of the 11 SEED level 2 nitrogen pathway annotations showed a significantly different relative abundance between sample types, including dissimilatory nitrite reductase, nitrate and nitrite ammonification, and nitrogen fixation (Table 1, Figure 1).

**Table 1:** **Average proportion of reads annotation to each SEED subsystems-based functional annotation (level 2) function from nitrogen metabolism with their standard deviations in parentheses**.

	**Oil seep**	**Oil spill**	**Uncontaminated**
Allantoin utilization	0.034 (0.008)	0.082 (0.024)	0.232 (0.123)
Ammonia assimilation	0.44 (0.007)	0.313 (0.029)	0.272 (0.227)
Denitrification	0.107 (0.031)	0.122 (0.010)	0.111 (0.034)
Dissimilatory nitrite reductase^*^	0.059 (0.005)	0.019 (0.006)	0.016 (0.010)
Nitrate and nitrite ammonification^*^	0.206 (0.021)	0.299 (0.016)	0.215 (0.074)
Nitrogen fixation^*^	0.055 (0.025)	0.136 (0.013)	0.153 (0.056)

* *p-value < 0.05*.

**Figure 2:**
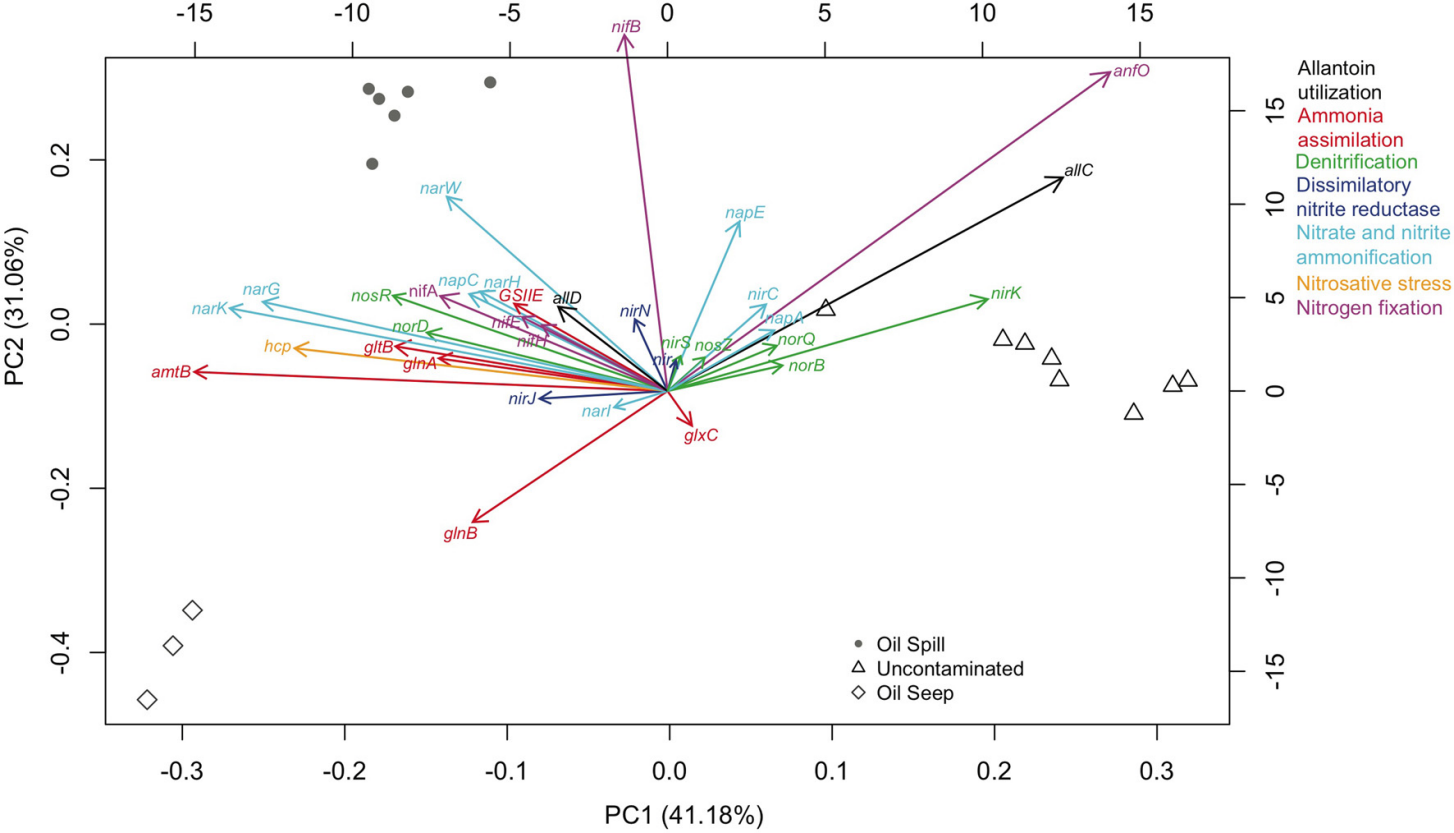
**Principal Components Analysis (PCA) of the relative abundance of nitrogen pathway gene annotation shared (33 genes) between the three samples types [oil spill (circles), oil seep (diamonds), and uncontaminated (triangles)]**. Genes on the graph are given abbreviated names, but their full names and associated functional groups are given in Supplementary Table 2. The gene abundance loading values are given by the bottom and left axes. Samples from each group shown as shapes in gray with axes scores given by the top and right axes. Gene functions are colored by their level 2 annotation as specified at the right outer margin of the graph. The variance accounted for by each component is listed on each axis.

A principal component analysis was performed using only the 33 genes that were present across all sample types, with the SEED level 2 hierarchical gene annotation used to demonstrate which genes showed the greatest differentiation between sample types (Figure 2). The first two principal components account for 72.78% of the variance, and while the variance and the distribution of the sample types was due to multiple factors, the influence of key genes in differentiation of sample types was evident. For example, an abundance of nitrogen regulatory protein P-II (*glnB*) in seep samples, respiratory nitrate reductase (alpha, beta, delta, and gamma chain; *narG, narH, narW* and *narI*, respectively) and nitrogenase (alpha and beta chain; *nifA* and *nifB*, respectively) in DWH spill samples, and Cu-nitrite reductase (*nirK*), AnfO protein (*anfO*), and allantoate amidohydrolase (*allC*) in uncontaminated sediments played a considerable role in the differentiation of the 3 sample types (Figure 2).

### The predicted relative turnover for nitrogen metabolites was significantly different between the 3 groups

The relative abundance of individual key genes showed differential responses (Figures 1, 2) across the gradient of hydrocarbon exposure time (seep>DWH spill>uncontaminated); therefore PRMT was used to infer how these differential relative abundances could be combined to predict relative metabolite turnover for the different nitrogen pathways. The KEGG nitrogen metabolism reference pathway includes 21 metabolites. The PRMT scores were used to infer whether these metabolites were relatively consumed or accumulated in each group; the PRMT score is positive if the metabolite is being consumed, and negative if it is being accumulated, with the magnitude an indication of the relative level of this metabolism. The overall “pathway flow” (the difference between predicted “net consumption” and “net production” for all metabolites in a pathway) was positive for the three sample types. A positive net pathway flow suggests that overall more nitrogen metabolites were being consumed than accumulated. The difference in “pathway flow” between the sample types was not significantly different; however, the highest “pathway flow” score was found in the oil seeps (PRMT “net difference” score [PRMT_diff_] = 11.35), followed by the DWH spill samples (PRMT_diff_ = 10.77) and uncontaminated sediments (PRMT_diff_ = 9.56). When the average “net accumulation” (negative PRMT) scores were summed per group, the difference between sample types was significantly different (*p* = 0.02) and 2-fold higher in the oil seep samples (PRMT “net production” [PRMT_*p*_] = −14.39) compared to the DWH spill (PRMT_*p*_ = −8.08) and uncontaminated sediments (PRMT_*p*_ = −7.15). For the specific metabolites in the pathway, there were also numerous significant differences between sample types (Table 2). Specifically, nitrate was predicted to be significantly more consumed in the oil seep (mean = 5.1, *SD* = 2.73) and DWH spill samples (mean = 2.98, *SD* = 3.41), compared to uncontaminated sediments, where it was predicted to be more significantly accumulated (mean = −0.75, *SD* = 0.73; *p*-value < 0.01). Meanwhile, nitrite was predicted to be significantly more accumulated in the oil seep (mean = −3.28, *SD* = 0.64) and DWH spill samples (mean = −1.99, *SD* = 1.67), while being relatively consumed in the uncontaminated sediments (mean = 0.37, *SD* = 0.58; *p*-value < 0.01). Genes annotating to nitrosative stress were only found in the hydrocarbon contaminated sediments, which is supported by the prediction that nitric oxide was significantly more accumulated in both the oil seep (mean = −2.3, *SD* = 1.27) and oil spill (mean = −1.1, *SD* = 0.31) compared to the uncontaminated sediments where it was relatively consumed (mean = 0.45, *SD* = 0.61). There was also a relative increase in the consumption of ammonia in the oil seep group, although this difference was not statistically significant (Table 1). In addition, nitrile was predicted to be significantly consumed (*p*-value < 0.001) in the oil seep (mean = 2.11, *SD* = 0.59) and the DWH spill samples (0.72, *SD* = 0.14), while being relatively accumulated in the uncontaminated sediments (mean = −0.34, *SD* = 0.33).

**Table 2:** **Nitrogen metabolism associated metabolites' mean (and standard deviation in parentheses) PRMT scores**.

**Metabolite**	**Oil Seep**	**Oil Spill**	**Uncontaminated**
	**(*N* = 3)**	**(*N* = 6)**	**(*N* = 8)**
NH_3_	0.41 (0.08)	−0.11 (0.31)	−0.27 (0.67)
Nitrite^**^	−3.28 (0.64)	−1.99 (1.67)	0.37 (0.58)
Carbamoyl phosphate^*^	0.047 (0.32)	−1.28 (0.36)	−1.1 (0.43)
Nitrate^**^	5.1 (2.73)	2.98 (3.41)	−0.75 (0.73)
Formamide^**^	2.59 (0.6)	1.84 (0.52)	−0.69 (0.79)
Nitric oxide^**^	−2.3 (1.27)	−1.11 (0.31)	0.45 (0.61)
Nitrogen^**^	0.42 (2.11)	−0.75 (0.62)	3.88 (2.88)
Nitrile^***^	2.11 (0.59)	0.72 (0.14)	−0.34 (0.33)
Nitrous oxide^*^	−0.86 (1.57)	0.29 (0.17)	−0.13 (0.14)
alpha-amino acid	0.39 (0.12)	0.45 (0.33)	0.33 (0.54)
L-aspartate^**^	1.36 (0.52)	0.46 (0.71)	−0.76 (0.44)
L-glutamine^*^	0.57 (0.07)	0.07 (0.08)	−0.11 (0.07)
CO_2^*^_	−0.4 (0.21)	−0.08 (0.46)	0.45 (0.54)
L-glutamate	−0.38 (0.11)	−0.39 (0.21)	−0.11 (0.51)
Glycine^*^	0.23 (0.13)	0.8 (0.41)	−0.001 (0.56)
Formate	−1.26 (0.66)	−0.31 (0.57)	−0.45 (0.94)
L-asparagine	−0.65 (0.64)	−0.26 (0.59)	0.58 (2.38)
Hydroxylamine	0.61 (3.21)	−0.66 (1.99)	−0.32 (1.20)
Amide	0.28 (0.54)	0.88 (0.74)	0.45 (0.62)
Amine	1.93 (0.35)	1.76 (1.24)	0.23 (1.35)
Cyclic amidines	0.39 (0.36)	0.29 (0.06)	1.03 (1.63)

* p-value < 0.05,

** p-value < 0.01,

*** p-value < 0.001.

*Positive values denote consumption and negative values denote accumulation of the metabolite*.

### PRMT scores for nitrogen pathway metabolites show significant correlations with in situ biogeochemical measurements between oil spill and uncontaminated sediment samples in the gulf of Mexico

The samples collected from the Gulf of Mexico were analyzed in more detail for significant correlations to the available biochemical data (Mason et al., 2014). PRMT scores for dinitrogen showed a significant positive correlation with measured concentrations of *in situ* total nitrogen (*p* < 0.05, corr = 0.55). In addition, a number of the other PRMT scores had significant correlations with total sulfur, total carbon, total nitrogen, dissolved nitrate, total ammonium, dissolved inorganic nitrogen, and dissolved phosphate (Table 3). The PRMT scores for nitrite had a significant negative correlation with total carbon (*p* < 0.01, corr = −0.58), which suggests that when there is more carbon there is a significant accumulation of nitrite. In addition, L-aspartate had a significant correlation (*p* < 0.05, corr = 0.53) with total hydrocarbon concentration.

**Table 3:** **Pearson correlations of metadata from 14 metagenome samples (Mason et al., 2014) with predicted relative nitrogen metabolite PRMT scores and their associated *p*-values**.

	**Total hydrocarbons**	**Total sulfur**	**Total carbon**	**Total nitrogen**	**Dissolved nitrate**	**Total ammonium**	**DIN**	**Dissolved phosphate**	**Sum of PAH**
NH_3_	−0.02	−0.01	0.21	−0.45	0.11	0.10	0.08	0.002	−0.04
Nitrite	−0.45	−0.66^**^	−0.58^**^	0.07	−0.48	−0.49	−0.52	−0.28	−0.37
Carbamoyl phosphate	−0.001	0.04	−0.42	0.41	−0.37	−0.19	−0.22	−0.29	0.07
Nitrate	0.42	0.64^**^	0.55^*^	0.02	0.45	0.48	0.50	0.26	0.39
Formamide	0.47	0.41	0.78^***^	−0.45	0.57^*^	0.44	0.49	0.45	0.33
Nitric oxide	−0.45	−0.47	−0.67^**^	0.34	−0.49	−0.42	−0.47	−0.32	−0.37
Nitrogen	−0.19	−0.21	−0.46	0.55^*^	−0.11	−0.45	−0.41	−0.34	−0.20
Nitrile	0.43	0.46	0.72^**^	−0.46	0.49	0.52^*^	0.54^*^	0.45	0.30
Nitrous oxide	0.44	0.44	0.57^*^	−0.41	0.51	0.49	0.51	0.57^*^	0.33
alpha-Amino acid	0.19	0.24	0.05	0.28	−0.09	−0.09	−0.09	−0.07	0.16
L-aspartate	0.53^*^	0.39	0.66^**^	−0.16	0.52^*^	0.23	0.29	0.29	0.47
L-glutamine	−0.40	−0.06	−0.13	−0.005	−0.21	0.04	0.002	−0.33	−0.39
CO_2_	0.10	0.13	−0.42	0.50	−0.29	−0.35	−0.40	−0.28	0.13
L-glutamate	0.36	0.19	0.49	−0.46	0.45	0.38	0.45	0.44	0.21
Glycine	0.47	0.51	0.56^*^	0.08	0.58^*^	0.28	0.33	0.37	0.45
Formate	0.17	−0.08	0.37	0.04	0.58^*^	0.001	0.11	0.29	0.23
L-asparagine	−0.08	−0.15	−0.24	0.28	−0.07	−0.15	−0.11	−0.11	0.002
Hydroxylamine	−0.37	0.05	−0.15	0.11	0.33	0.33	0.39	0.15	−0.56
Amide	0.43	0.71^**^	0.32	0.42	0.33	0.30	0.34	0.19	0.41
Amine	0.29	0.07	0.48	−0.04	0.22	0.07	0.09	0.18	0.43
Cyclic amidines	0.05	−0.19	−0.33	0.39	−0.14	−0.33	−0.31	−0.05	0.14

* p-value < 0.05,

** p-value < 0.01,

*** *p-value < 0.001*.

## Discussion

Here we present evidence of the impact of oil contamination, including comparisons of short-term vs. long-term duration of exposure, on nitrogen metabolism in marine sediments. Oil contaminated and uncontaminated sediment samples collected after the DWH spill in the Gulf of Mexico were compared to samples collected from natural oil seeps from the Santa Barbara Channel. Genes and pathways involved in the nitrogen cycle were annotated from metagenomic sequencing data and used to explore differences in the relative abundance of specific genes and to predict relative nitrogen metabolite turnover potential between the 3 sample types. These sample types come from disparate regions (e.g., Gulf of Mexico vs. Santa Barbara Channel), thus numerous other geochemical and physical factors could have played a role in the observed trends in nitrogen metabolism between these environments. However, this study suggests that the selective pressure of oil contamination contributes a significant role toward shaping the functional diversity of these community processes. In addition, we expand on an analysis of metagenome data (Mason et al., 2014) and show that this analysis can be useful for exploring the impacts of hydrocarbon contamination on nitrogen cycling in other contaminated environments.

Studies of the relative abundances of specific genes may not be the best way to study complex, multi-branching metabolic pathways. To overcome this limitation, we used PRMT to better capture the emergent property of the multiphasic gene abundance profiles that make up a metabolic pathway. The PRMT approach captures the relative metabolic changes across an observed assemblage of genes, and therefore the relative abundances of genes and their corresponding metabolic pathways are taken in proportion to each other (Larsen et al., 2011). The predicted “net pathway flow” suggests that overall more nitrogen metabolites were consumed in each sample type than were accumulated, with this value being greatest in the oil seep samples. While this might seem to infer a system mass balance bias, as the data used for predictions is static, these inferences cannot be used to infer mass potential. For those metabolites that are predicted to be accumulated, there was a two-fold increase in the oil seep samples compared to DWH spill and uncontaminated sediment samples. In addition, the specific metabolites that were predicted to accumulate in the contaminated samples were different from those in the uncontaminated samples, which may represent shifts in nitrogen cycling processes in sediments exposed to hydrocarbon saturation.

The metabolites that were significantly different between the three groups, i.e., nitrate, nitrite, and nitric oxide had a common trend in which the oil seep samples had the highest consumption and accumulation, followed by the DWH spill samples, and finally the uncontaminated samples, where the values were often close to 0; suggesting that the pathways involved in consumption and accumulation of nitrogen were balanced. Dinitrogen was an exception in that the uncontaminated sediments had a three-fold higher predicted consumption than in the contaminated samples. This was supported by the relative abundance of genes involved in diazotrophy (nitrogen fixation), which were most abundant in uncontaminated sediments.

The sum of predicted metabolite turnover scores for all nitrogen metabolism pathways evidenced an increase in denitrification processes either through canonical denitrification or anammox, as nitrite and nitric oxide were predicted to significantly accumulate and nitrate was predicted to be consumed by microbial metabolism in contaminated samples. It is more likely that this evidence could be interpreted as relating to canonical denitrification, despite the relatively higher abundance of anammox pathway specific genes related to hydrazine production and ammonium assimilation in seep and spill compared to uncontaminated samples.

The oil spill sediments from the Gulf of Mexico were collected ~3 months after the Deepwater Horizon's Macondo well was capped, thus giving them an active exposure time between 3 and 5 months, if we assume the absence of natural oil seeps near these sites. By contrast, the oil seep samples from the Santa Barbara Channel were actively exposed to petroleum for more than 11,000 years (Hornafius et al., 1999) and samples were taken directly from the seep head. The difference in the time of exposure to hydrocarbon pollution in the oil-contaminated sediments could thus account for differences in the predicted turnover of nitrogen metabolites. Additionally, significant differences in the composition of the oil from both sites (Hornafius et al., 1999; Reddy et al., 2011), may also have influenced the observed compositional differences the microbial communities (Hawley et al., 2014; Mason et al., 2014). Depth of sample collection from the two different environments may also have affected nitrogen cycling as shown in several studies (Engström et al., 2009; Trimmer et al., 2013), however, this trend is probably due to differences in the physicochemical properties of different sites, as has been shown for sites at different distances from shore (Herbert, 1999; Dalsgaard et al., 2005; Zhu et al., 2010). Despite these geographic, depth, and oil composition differences, there were surprising similarities in the response of metabolic turnover to hydrocarbon contamination, suggesting that oil contamination results in a predictable metabolic response despite differences in the affected ecosystems. It is suggested that a topic for future research might be using PRMT on genetic data generated from oil exposed laboratory enrichments or environmental samples through time, to aid in unraveling the relationship between nitrogen cycling and microbial oil remediation.

Nitrite consumption showed a significant negative correlation to the concentration of total carbon in the Gulf of Mexico sediments. The decreased availability of reactive carbon and a high concentration of organic carbon in extremely deep benthic environments would favor the dominance of anammox over denitrification (Thamdrup and Dalsgaard, 2002; Engström et al., 2005), which would therefore lead to direct oxidation of ammonia to dinitrogen reducing nitrite consumption. This would however lead to a nitrogen limited environment, which could be supplanted by the oxidation of organic matter by sulfur reducing bacteria (Canfield et al., 2010); this is potentially supported by significant correlations between the concentration of sulfur and the accumulation of nitrate and nitrite.

To summarize, there is evidence from the PRMT analysis for a shift in the metabolic flow of nitrogen to the denitrification pathways, potentially including the anammox pathway, in hydrocarbon-contaminated sediments (both DWH spill and natural oil seep). Changes in metabolites in the anammox pathway were positively correlated with hydrocarbon concentration, although these were not statistically significant, potentially due to the small sample sizes and confounding environmental factors. The relative abundance of genes related to anammox associated hydrazine metabolism were also greatest in the seep samples that were predicted to have been exposed to hydrocarbons for ~11,000 years.

Marine sediments are very important sites for microbially mediated nitrogen transformation, providing a link between organic matter degradation and nutrient regeneration, essentially supporting primary productivity in the oceans. Exploring factors that significantly influence this process are vital for providing relevant data to propagate system scale models of how basin processes, such as nitrogen cycling and primary productivity in marine sediments, can influence regional and global climate.

### Conflict of interest statement

The authors declare that the research was conducted in the absence of any commercial or financial relationships that could be construed as a potential conflict of interest.
