# Supplementary material for: The microbial nitrogen cycling potential is impacted by polyaromatic hydrocarbon pollution of marine sediments
Source: Front Microbiol. 2014 Mar 25;5:108. doi: 10.3389/fmicb.2014.00108 (PMC3971162; doi:10.3389/fmicb.2014.00108)
Supplement: Supplementary file 1 [file DataSheet1.DOCX]

Supplementary Table 1. Metadata for three oil seep samples from the Santa Barbara Channel.

| Sample ID | Depth | Latitude (North) | Longitude (West) | Date Collected |
| --- | --- | --- | --- | --- |
| 4537092.3 | 65 | 34.3751 | -119.8532 | 6/19/09 |
| 4537093.3* | 79.4 | 34.39192 | -119.84578 | 6/19/09 |
| 4537094.3 | 46.7 | 34.3751 | -119.8532 | 6/19/09 |

*Sample published in Hawley et al., 2014
