# Supplementary material for: The microbial nitrogen cycling potential is impacted by polyaromatic hydrocarbon pollution of marine sediments
Source: Front Microbiol. 2014 Mar 25;5:108. doi: 10.3389/fmicb.2014.00108 (PMC3971162; doi:10.3389/fmicb.2014.00108)
Supplement: Supplementary file 2 [file DataSheet2.DOCX]

Supplementary Table 2. Gene, annotations, and associated functional groups represented in Figure 2.

| Gene | Original Annotation Name | Functional Group |
| --- | --- | --- |
| *allC* | "Allantoate amidohydrolase (EC 3.5.3.9)" | Allantoin utilization |
| *allD* | "Ureidoglycolate dehydrogenase (EC 1.1.1.154)" | Allantoin utilization |
| *GSIIE* | "Glutamine synthetase type II, eukaryotic (EC 6.3.1.2)" | Ammonia assimilation |
| *amtB* | "Ammonium transporter" | Ammonia assimilation |
| *gltB* | "Glutamate synthase NADPH large chain (EC 1.4.1.13)" | Ammonia assimilation |
| *glxC* | "Glutamate synthase NADPH putative GlxC chain (EC 1.4.1.13)" | Ammonia assimilation |
| *glnA* | "Glutamine synthetase type I (EC 6.3.1.2)" | Ammonia assimilation |
| *glnB* | "Nitrogen regulatory protein P-II" | Ammonia assimilation |
| *nirK* | "Copper-containing nitrite reductase (EC 1.7.2.1)" | Denitrification |
| *nirS* | "Cytochrome cd1 nitrite reductase (EC:1.7.2.1)" | Denitrification |
| *norD* | "Nitric oxide reductase activation protein NorD" | Denitrification |
| *norQ* | "Nitric oxide reductase activation protein NorQ" | Denitrification |
| *norB* | "Nitric-oxide reductase subunit B (EC 1.7.99.7)" | Denitrification |
| *nosR* | "Nitrous oxide reductase maturation protein NosR" | Denitrification |
| *nosZ* | "Nitrous-oxide reductase (EC 1.7.99.6)" | Denitrification |
| *nir* | "Cytochrome cd1 nitrite reductase (EC:1.7.2.1)" | Dissimilatory nitrite reductase |
| *nirJ* | "Heme d1 biosynthesis protein NirJ" | Dissimilatory nitrite reductase |
| *nirN* | "Nitrite reductase associated c-type cytochorome NirN" | Dissimilatory nitrite reductase |
| *napC* | "Cytochrome c-type protein NapC" | Nitrate and nitrite ammonification |
| *narK* | "Nitrate/nitrite transporter" | Nitrate and nitrite ammonification |
| *nirC* | "Nitrite transporter from formate/nitrite family" | Nitrate and nitrite ammonification |
| *napE* | "Periplasmic nitrate reductase component NapE" | Nitrate and nitrite ammonification |
| *napA* | "Periplasmic nitrate reductase precursor (EC 1.7.99.4)" | Nitrate and nitrite ammonification |
| *narG* | "Respiratory nitrate reductase alpha chain (EC 1.7.99.4)" | Nitrate and nitrite ammonification |
| *narH* | "Respiratory nitrate reductase beta chain (EC 1.7.99.4)" | Nitrate and nitrite ammonification |
| *narW* | "Respiratory nitrate reductase delta chain (EC 1.7.99.4)" | Nitrate and nitrite ammonification |
| *narI* | "Respiratory nitrate reductase gamma chain (EC 1.7.99.4)" | Nitrate and nitrite ammonification |
| *anfO* | "AnfO protein, required for Mo- and V-independent nitrogenase" | Nitrogen fixation |
| *nifA* | "Nitrogenase (molybdenum-iron) alpha chain (EC 1.18.6.1)" | Nitrogen fixation |
| *nifH* | "Nitrogenase (molybdenum-iron) reductase and maturation protein NifH" | Nitrogen fixation |
| *nifB* | "Nitrogenase (vanadium-iron) beta chain (EC 1.18.6.1)" | Nitrogen fixation |
| *nifE* | "Nitrogenase FeMo-cofactor scaffold and assembly protein NifE" | Nitrogen fixation |
| *hcp* | "Hydroxylamine reductase (EC 1.7.-.-)" | Nitrosative stress |
